# Factors that increase the rate of periprosthetic dislocation after reverse shoulder arthroplasty

**DOI:** 10.1186/s42836-023-00214-2

**Published:** 2023-12-02

**Authors:** Chethan Reddy, Nikit Venishetty, Hunter Jones, Varatharaj Mounasamy, Senthil Sambandam

**Affiliations:** 1 Perelman School of Medicine, University of Pennsylvania, 3400 Civic Center Blvd, Philadelphia, PA 19104 USA; 2 Paul L. Foster School of Medicine, Texas Tech University Health Sciences Center, 5001 El Paso Dr, El Paso, TX 79905 USA; 3 University of Texas Southwestern, 5323 Harry Hines Blvd, Dallas, TX 75390 USA; 4 Department of Orthopedics, University of Texas Southwestern, Dallas VAMC, 4500 South Lancaster Road, Dallas, TX 75216 USA

**Keywords:** Perioperative complications, Periprosthetic dislocation, Reverse shoulder arthroplasty

## Abstract

**Introduction:**

Reverse shoulder arthroplasty (RSA) is considered one of the greatest technological innovations in shoulder reconstruction surgery, as evidenced by the fact its growth rate of usage is greatest among all shoulder arthroplasties. However, like all arthroplasties, a post-surgical complication often arises. One of these complications, periprosthetic dislocation (PPD), requires revision and poses, therefore, a burden on both patients and healthcare providers. While PPD is understood to be a complication of RSA, it is unclear to what extent certain risk factors and co-morbidities predispose patients to post-RSA PPD. The purpose of this study was to identify and evaluate the impact of specific risk factors and co-morbidities that contribute to the development of PPD following RSA.

**Methods:**

In this retrospective study, we used the Nationwide Inpatient Sample (NIS) database from 2016–2019 to analyze the prevalence and impact of various risk factors and co-morbidities on the incidence of PPD following RSA. A univariate and subsequent multivariate logistic regression model was made to provide a descriptive association between variables that impact the rates of PPD after RSA.

**Results:**

The NIS database identified 59,925 patients, 1,000 of whom experienced a PPD while the remaining 58,825 were placed in the non-PPD group (controls). The PPD group consisted predominantly of females (53.10%) and Caucasians (86.30%). There was a higher incidence of tobacco-related disorders (*P* = 0.003), obesity (*P* < 0.001), morbid obesity (*P* < 0.001), liver cirrhosis (*P* < 0.001), and Parkinson’s disease (PD) (*P* < 0.001) in PPD patients compared to controls. Young patients had a 1.89-fold increased odds (OR: 1.89, 95% CI [1.58, 2.26], *P* < 0.001), patients with tobacco-related disorders had decreased odds (OR: 0.80, 95% CI [0.67, 0.97], *P* = 0.02), morbidly obese patients had 1.50 times the odds (OR: 1.50, 95% CI [1.14, 1.97]), liver cirrhosis patients had 2.67-fold increased odds (OR: 2.67, 95% CI [1.55, 4.60], *P* < 0.001), and Parkinson’s disease patients had 2.66 times the odds (OR: 2.66, 95% CI [1.78, 3.96], *P* < 0.001) to develop PPD following RSA compared to patients who did not have the corresponding condition.

**Conclusions:**

Patients with specific risk factors and co-morbidities are predisposed to developing PPD after RSA. Risk factors that were found to be associated with a higher incidence of PPD are gender (female), race (Caucasian), and age (young patients). Analysis revealed the history of tobacco-related disorder, obesity, morbid obesity, liver cirrhosis, and Parkinson’s disease increased the odds of developing PPD following RSA. These findings can inform both healthcare providers and patients to improve RSA surgical outcomes and tailor post-surgery recovery programs to fit the patient’s needs.

## Introduction

Reverse shoulder arthroplasty (RSA) is a surgical procedure that treats disease, degeneration, and trauma of the glenohumeral joint introduced in the 1970s [1]. RSA differs from conventional shoulder arthroplasties in that it “reverses” the anatomy of the glenohumeral joint, placing a socket in the humerus and a prosthetic ball on the glenoid [1, 2]. RSAs have recently become more popular, nearly tripling in incidence over the past decade [3]. The major advantage of RSA is that it not only improves shoulder joint stability but also improves range of motion [1].

RSA, like all shoulder arthroplasties, is not without complications. Infection, aseptic loosening, notching, fracture, and dislocation are relatively common complications arising from this procedure [1–3]. Other studies reported periprosthetic dislocation (PPD) was the second leading complication of RSA, occurring at rates from 2.1% to 4.7% [4, 5]. A previous study found that revision surgery following primary RSA dislocation was only successful in 50% of cases, begetting further revision surgery [6]. It is apparent that these revision surgeries burden both the patient and the healthcare system and should be avoided/mitigated if possible.

Increasingly, patients requiring RSA often have comorbid conditions that have previously been reported to increase the risk for complications such as infection and loosening [4]. However, there is very limited information as to what factors influence the rates of PPD in RSA. The purpose of this study was to analyze a large patient database to understand the extent to which specific risk factors and comorbidities impact the odds of developing a PPD following primary RSA.

## Methods

### NIS database acquisition

The Nationwide (or National) Inpatient Sample (NIS) contains information on more than 7,000,000 hospital stays and is the biggest all-payer, publicly accessible inpatient care database in the USA [7]. The NIS database was used to get information on patients who received primary RSA between 2016 and 2019 in the USA. Among this sample, patients who experienced a periprosthetic dislocation were identified. Due to the enormous size of the sample, it offers the perfect information to create national/regional estimations. It also makes it possible to analyze unusual pathologies, uncommon treatments, and particular demographics. The diseases are categorized by using the International Classification of Disease-Tenth Revision, Clinical Modification/Procedure Coding System (ICD-10-CM/PCS) in the NIS database version that was available between 2016 and 2019.

### Data gathering

Our study did not require Institutional Review Board (IRB) approval because the data in the NIS database is de-identified and open to the public. The International Classification of Diseases (ICD-10) codes were used to identify every patient who received RSA. Parameters including patient demographics (gender, race, age) and comorbidities and preoperative factors (tobacco-related disorders, elective admission, obesity, super obesity, morbid obesity, diabetes with or without complications, liver cirrhosis, Parkinson’s disease, dialysis, organ transplant) were identified to assess rates of periprosthetic dislocation (PPD) following RSA. Morbid obesity was defined as a body mass index (BMI) greater than 40 kg/m^2^, and super obesity was defined as a BMI over 50 kg/m^2^. According to the ICD-10 codes, young patients were defined as patients less than 65 years of age, and not-young patients were defined as patients older than 65 years of age.

### Statistical analysis

Statistical analysis was performed using SPSS version 27.0 (IBM; Armonk, NY, USA). The *t*-test and the Chi-square test were employed, respectively, to assess numerical and categorical variables with an occurrence of less than 5. Univariate analysis was conducted, and subsequent multivariable logistic regression analysis was also performed. The odds ratios (measured as ratios between the relative incidence in the PPD group and non-PPD group) and 95% confidence intervals were calculated for the diverse outcome parameters. Any *P-value* less than 0.05 was deemed statistically significant for the study’s purposes.

## Results

### Patient demographic data analysis

59,925 patients were identified in the NIS database during the study period. Of these patients, 1,000 patients (1.7%) suffered periprosthetic dislocation (PPDs) while the remaining 58,925 patients were assigned to the control group. The demographic data for the groups are included in Table 1. Females comprised a larger proportion of the PPD (53.10%) and control groups (60.69%) compared to males (*P* < 0.001). The PPD group had a significantly higher proportion of obese (PPD: 24.60%, Control: 19.89%, *P* < 0.001), super obese (PPD: 1.60%, Control: 0.84%, *P* = 0.014), and morbidly obese patients (PPD: 12.60%, Control: 8.02%, *P* < 0.001). No significant differences were found between the PPD and control groups within a given patient ethnicity. However, Caucasians accounted for a higher proportion of the PPD and control groups compared to African-American, Hispanic, Asian, and Native American patients.

**Table 1:** Demographic characteristics of periprosthetic dislocation patients and the control group patients

	Periprosthetic Dislocation Group (*n* = 1,000)	Control Group (*n* = 58,925)	Significance
Age Binomial	244 (24.40%)	19,559 (33.20%)	**<0.001**
Female	531 (53.10%)	35,761 (60.69%)	**<0.001**
Minority	88 (9.25%)	6,548 (11.56%)	**0.015**
Race			
Caucasian	863 (86.30%)	50,079 (84.99%)	0.13
African American	38 (3.80%)	2,504 (4.25%)	0.27
Hispanic	35 (3.50%)	2,630 (4.46%)	0.08
Asian	^a^ (0.60%)	335 (0.57%)	0.51
Native American	^a^ (0.20%)	204 (0.35%)	0.33

Bold values indicate a statistically significant result (*P* < 0.05)

^a^Numbers between 1 and 10 were not reported per the healthcare cost and utilization project data agreement

### Univariate analysis of patient co-morbidities and other factors

The prevalence of tobacco-related disorders was significantly higher in the control group (16.1%) than in the PPD group (12.9%, *P* = 0.003). Additionally, control group patients were significantly more likely to electively admit to the clinic than PPD patients (92.6% vs. 90.1%, *P* < 0.001). There was no significant difference in diabetes characteristics between the two groups (Table 2). A significantly higher fraction of PPD patients suffered from liver cirrhosis (PPD: 1.4% vs. control: 0.5%, *P* < 0.001) or Parkinson’s Disease (PPD: 2.7% vs. control: 1.1%, *P* < 0.001) in comparison to controls. No significant differences were found between PPD and control group patients with CKD or who underwent dialysis, other crystal arthroplasty, or organ transplant.

**Table 2:** Univariate analysis periprosthetic dislocation patients and the control group patients

Variable	Periprosthetic Dislocation Group (*n* = 1,000)	Control Group (*n* = 58,925)	Significance
Tobacco-Related Disorder	129 (12.90%)	9,515 (16.15%)	**0.003**
Elective Admission	899 (90.10%)	54,505 (92.50%)	**0.01**
Obesity	246 (24.60%)	11,718 (19.89%)	**<0.001**
Super Obesity	16 (1.60%)	497 (0.84%)	**0.014**
Morbid Obesity	126 (12.60%)	4,724 (8.02%)	**<0.001**
Diabetes Without Complications	142 (14.20%)	8,515 (14.45%)	0.43
Diabetes With Complications	^a ^(0.10%)	117 (0.20%)	0.41
Liver Cirrhosis	14 (1.40%)	293 (0.50%)	**<0.001**
Parkinson’s Disease	27 (2.70%)	663 (1.13%)	**<0.001**
CKD	82 (8.20%)	4,687 (7.95%)	0.41
Dialysis	^a^ (3.00%)	97 (0.16%)	0.23
Organ Transplant	^a^ (0.40%)	152 (0.26%)	0.26

Bold values indicate a statistically significant result (*P* < 0.05)

^a^Numbers between 1 and 10 were not reported per the healthcare cost and utilization project data agreement. CKD: chronic kidney disease

### Multivariate analysis

Young patients had 1.89 times the odds to have a PPD than their non-young counterparts (95% CI [1.58, 2.26], *P* < 0.001). Patients with a tobacco-related disorder were placed at 0.80 odds of having a PPD as those without such a disorder (OR: 0.80, 95% CI [0.67, 0.97], *P* = 0.02). Moreover, females had lower odds to have a PPD than non-females, (OR: 0.73, 95% CI [0.64, 0.83], *P* < 0.001). Similarly, the odds of PPD in patients undergoing elective RSA was less (OR: 0.68, 95% CI [0.55, 0.84], *P* < 0.001) compared to the emergency RSA. However, there were significantly higher odds of having a PPD in morbidly obese vs. non-morbidly obese patients (OR: 1.50, 95% CI [1.14, 1.97], *P* = 0.004). Patients with liver cirrhosis, compared to those without liver cirrhosis, were found to have significantly higher odds of having a PPD (OR: 2.67, 95% CI [1.55, 4.60], *P* < 0.001). A similar finding was seen in patients with PD vs. patients without PD (OR: 2.66, 95% CI [1.78, 3.96], *P* < 0.001). No significant differences were found in the odds of having a PPD for obese vs. non-obese, super obese vs. non-super obese, or minority vs. non-minority patients (Table 3).

**Table 3:** Multivariate analysis: periprosthetic dislocation patients and the control group patients

Variable	Periprosthetic Dislocation Group (*n* = 1,000)	Control Group (*n* = 58,925)	Odds Ratio (Periprosthetic dislocation/Control Group)	Odds Ratio 95% Confidence Interval	Significance
Young		**-**	1.89	(1.58, 2.26)	**<0.001**
Obesity	246 (24.60%)	11,718 (19.89%)	1.02	(0.83, 1.24)	0.88
Tobacco-related Disorder	129 (12.90%)	9,515 (16.15%)	0.80	(0.67, 0.97)	**0.02**
Female	531 (53.10%)	35,761 (60.69%)	0.73	(0.64, 0.83)	**<0.001**
Elective Admission	899 (90.10%)	54,505 (92.50%)	0.68	(0.55, 0.84)	**<0.001**
Super Obesity	16 (1.60%)	497 (0.84%)	1.20	(0.69, 2.09)	0.51
Morbid Obesity	126 (12.60%)	4,724 (8.02%)	1.50	(1.14, 1.97)	**0.004**
Liver Cirrhosis	14 (1.40%)	293 (0.50%)	2.67	(1.55, 4.60)	**<0.001**
Parkinson’s Disease (PD)	27 (2.70%)	663 (1.13%)	2.66	(1.78, 3.96)	**<0.001**

Bold values indicate a statistically significant result (*P* < 0.05)

## Discussion

PPD is the leading complication of reverse shoulder arthroplasty (RSA), often occurring within 3 months after primary surgery, with an incidence rate of up to 10% [6, 8]. There has been a substantial increase in RSA procedures as their acceptance grows, so it is important to understand the most common RSA complication [9]. Previous work has demonstrated that there are prior risk factors that are significantly associated with PPD incidence [10]. In addition, PPD has been shown to be associated with THA [11, 12]. However, it is still unclear to what extent co-morbidities modify PPD risk. The purpose of the present study was to analyze the prevalence and impact of various risk factors and co-morbidities on the incidence of PPD following RSA in a large contemporary database in the USA. Previous findings have also suggested certain conditions in the risk of developing a PPD, and therefore the current study investigated various comorbidities [13].

Having a tobacco-related disorder was found to put patients at lower odds of developing a PPD, which seemed in contrast with findings that tobacco use might increase the risk for periprosthetic joint infection [14]. This finding might stem from the fact that smokers tend to have lower BMIs than non-smokers, and BMI is correlated with a higher incidence of post-arthroplasty complications, such as infection and dislocation [15].

Interestingly, obesity and super obesity had no significant effect on the risk of developing PPD. However, we found that morbid obesity (BMI > 40) significantly increased the odds of developing PPD. These findings on obesity and morbid obesity are supported by the literature. Kusin et al. demonstrated that obesity did not correlate with post-RSA dislocation events [15]. Moreover, meta-analyses only yielded mixed or inconclusive results when evaluating the relationship between obesity and PPD [16]. However, recent work by Sinkler et al. found a significant association between obesity, specifically a BMI of 38–40, and risk of PPD [17]. Research has found that a larger amount of enveloping adipose tissues increases the time the shoulder is exposed during operation, increasing the likelihood of subsequent dislocation, following RSA and other joint replacement surgeries [18, 19]. Moreover, it has been hypothesized that the heavier arms of morbidly obese patients can predispose the artificial joint to early wear and dislocation [16, 20]. Surgeons must take these considerations into account when planning RSA for morbidly obese patients to cut down operative time and inform patients with morbid obesity how to properly care for their prosthesis. Interestingly, our findings contradicted those that predominate in the literature regarding super obese patients (BMI > 50). Super obesity has been shown to be associated with a significant increase in the odds of developing PPD following RSA, although the research on this cohort is spare [21].

Parkinson’s disease (PD) was found to significantly increase the odds of PPD following RSA by 2.67 times. This finding is well-supported by the literature [22, 23]. It is believed that PD patients have an increased risk of PPD incidence for several reasons related to neuromuscular disease. Koch et al. found that PD patients developed glenohumeral subluxation following RSA because of reduced musculature and rehabilitation ability [22]. Moreover, Burrus et al. hypothesized that secondary causes related to PD symptomology such as rigidity/tremor, and osteoporotic bone potentially increase RSA dislocation risk in PD patients [22, 23]. These results should warn patients and healthcare providers to ensure proper management of patients with PD to minimize and mitigate PD-mediated RSA complications.

We further demonstrated that patients with liver cirrhosis were 2.66 times more likely to develop a PPD following RSA than patients without liver cirrhosis. Research has been scant regarding the association between liver cirrhosis and RSA dislocation. However, prior work confirmed a similar association between liver cirrhosis patients and total hip arthroplasty (THA). Tiberi et al. reported that liver cirrhosis patients had higher rates of THA dislocation in a database spanning 12 years [24, 25]. Liver cirrhosis puts patients at risk for deep prosthetic infection and subsequent loosening, which predisposes these patients at a higher risk for dislocation [24–26]. Liver cirrhosis must therefore not only be taken into account during surgery but also be properly included in post-treatment to mitigate future complications.

## Limitations

The limitations of our study stem particularly from the analysis of historical data. All the information on postoperative PPD collected from the NIS database was gathered only during the patient’s in-hospital stay. Therefore, the NIS database may not cover a large portion of dislocations, as previous work by Sinkler et al. found that the mean time to PPD following primary RSA was 60 months [17]. Additionally, it is probable that specific co-morbidities, risk factors, and perioperative events were overlooked because the present study mainly relied on the data from the NIS database. As previously mentioned, we could not gather information on certain important aspects, such as the surgical technique, implant type, perioperative anesthesia-related factors, medications used, etc. In addition, we included both elective and emergency RSAs in our analysis. Because emergent RSA is associated with a higher mortality rate, this aspect might have caused some underlying bias in our study’s selection process. Another limitation of our study is the inability to provide information regarding the various indications for RSA. We were only able to provide information regarding elective versus non-elective surgeries. However, the limited capacity of the NIS prevents us from providing a more comprehensive view of RSA. The NIS database’s vast sample size, however, offers incredibly valuable information that is ideal for evaluating and recognizing the preoperative risk factors and complications that may modify the risk of developing a PPD or mortality following RSA.

## Conclusion

Patients with specific risk factors and co-morbidities are predisposed to developing PPD after RSA. Patients with morbid obesity, Parkinson’s disease, and liver cirrhosis were found to be most associated with periprosthetic dislocation following reverse shoulder arthroplasty. These findings can inform both healthcare providers and patients to help them improve RSA surgical outcomes and tailor post-surgery recovery programs to fit the patient’s needs. These findings indicate that providers should regularly screen for these risk factors and appropriately optimize and tailor post-surgery recovery plans for patients to mitigate the overall post-RSA PPD rates.

## Data Availability

The datasets used and/or analyzed during the current study are available from the corresponding author on reasonable request.
